# Supplementary material for: How do health professionals face barriers? A quantitative approach to the adoption of evidence-based practices in mental health care
Source: Glob Ment Health (Camb). 2026 Feb 20;13:e52. doi: 10.1017/gmh.2026.10159 (PMC13112285; doi:10.1017/gmh.2026.10159)
Supplement: Félix-Romero et al. supplementary material [file S2054425126101599sup001.docx]

|  | **Tabla 6.**  *Standardized factor loadings of Facilitators and Barriers for mhGAP Adoption Questionnaire.* | | | | | | | | | | |
| --- | --- | --- | --- | --- | --- | --- | --- | --- | --- | --- | --- |
|  |  | **Standardized factor loadings** | ***X2*** | ***p*** | ***df*** | **CFI** | **TLI** | **RMSEA** | **SRMR** | α | |
| Facilitadores de material | | | | | | | | | | |  |
|  | Facilidad de los procedimientos de la guía mhGAP | 0.742 | 14.38 | 0.347 | 13 | 0.998 | 0.997 | 0.029 | 0.019 | 0.925 | |
|  | Pertinencia de los procedimientos de la guía mhGAP | 0.742 |  |  |  |  |  |  |  |  |  |
|  | Disponibilidad del material sobre la guía mhGAP | 0.642 |  |  |  |  |  |  |  |  |  |
|  | Claridad de la información de la guía mhGAP | 0.883 |  |  |  |  |  |  |  |  |  |
|  | Lenguaje de la guía entendible para profesionales de la salud | 0.916 |  |  |  |  |  |  |  |  |  |
|  | Disponibilidad de ejemplos prácticos en la guía mhGAP | 0.861 |  |  |  |  |  |  |  |  |  |
|  | Percepción de utilidad de la guía mhGP | 0.830 |  |  |  |  |  |  |  |  |  |
| Facilitadores de capacitación | | | | | | | | | | |  |
|  | Frecuencia con la que recibes capacitación en la guía mhGAP | 0.802 | 6.15 | 0.045 | 2 | 0.983 | 0.95 | 0.129 | 0.024 | 0.863 | |
|  | Práctica suficiente en la capacitación en la guía mhGAP | 0.899 |  |  |  |  |  |  |  |  |  |
|  | Modalidad presencial de la capacitación en la guía mhGAP | 0.721 |  |  |  |  |  |  |  |  |  |
|  | Modalidad a distancia de la capacitación en la guía mhGAP | 0.748 |  |  |  |  |  |  |  |  |  |
| Facilitadores de aplicación | | | | | | | | | | |  |
|  | Modalidad presencial de las intervenciones | 0.450 | 10.92 | 0.142 | 15 | 0.988 | 0.973 | 0.067 | 0.032 | 0.844 | |
|  | Modalidad a distancia de las intervenciones | 0.551 |  |  |  |  |  |  |  |  |  |
|  | Supervisión para la implementación de la guía mhGAP | 0.822 |  |  |  |  |  |  |  |  |  |
|  | Facilidad de comunicación con otros profesionales de la salud | 0.886 |  |  |  |  |  |  |  |  |  |
|  | Disponibilidad de servicios para la derivación de casos | 0.793 |  |  |  |  |  |  |  |  |  |
|  | Disponibilidad de medicamentos para la atención de casos | 0.670 |  |  |  |  |  |  |  |  |  |
| Barreras de aplicación | | | | | | | | | | |  |
|  | Tiempo disponible para implementar la guía mhGAP | 0.635 | 61.3 | 0.003 | 34 | 0.958 | 0.944 | 0.08 | 0.041 | 0.909 | |
|  | Dificultad de los procedimientos de la guía mhGAP | 0.612 |  |  |  |  |  |  |  |  |  |
|  | Pertinencia de los procedimientos de la guía mhGAP | 0.769 |  |  |  |  |  |  |  |  |  |
|  | Duración de las intervenciones | 0.760 |  |  |  |  |  |  |  |  |  |
|  | Modalidad presencial de las intervenciones | 0.692 |  |  |  |  |  |  |  |  |  |
|  | Modalidad a distancia de las intervenciones | 0.727 |  |  |  |  |  |  |  |  |  |
|  | Derivación de las personas a otros servicios | 0.784 |  |  |  |  |  |  |  |  |  |
|  | Implementación de intervenciones psicosociales | 0.813 |  |  |  |  |  |  |  |  |  |
|  | Implementación de intervenciones farmacológicas | 0.545 |  |  |  |  |  |  |  |  |  |
|  | Implementación de intervenciones comunitarias | 0.697 |  |  |  |  |  |  |  |  |  |
| Barreras del material | | | | | | | | | | |  |
|  | Disponibilidad del material sobre la guía mhGAP | 0.662 | 8.07 | 0.045 | 3 | 0.994 | 0.979 | 0.116 | 0.010 | 0.946 | |
|  | Pertinencia del material de la guía mhGAP para la población | 0.77 |  |  |  |  |  |  |  |  |  |
|  | Claridad de la información del material de la guía mhGAP | 0.934 |  |  |  |  |  |  |  |  |  |
|  | Lenguaje del material entendible para la persona | 0.975 |  |  |  |  |  |  |  |  |  |
|  | Lenguaje del material entendible para profesionales de la salud | 0.980 |  |  |  |  |  |  |  |  |  |
| Barreras de capacitación | | | | | | | | | | |  |
|  | Frecuencia con la que recibes capacitación en la guía mhGAP | 0.431 | 43.79 | 0.011 | 25 | 0.941 | 0.914 | 0.078 | 0.06 | 0.806 | |
|  | Duración de la capacitación en la guía mhGAP | 0.612 |  |  |  |  |  |  |  |  |  |
|  | Dificultad de los contenidos de capacitación en la guía mhGAP | 0.625 |  |  |  |  |  |  |  |  |  |
|  | Práctica insuficiente en la capacitación en la guía mhGAP | 0.819 |  |  |  |  |  |  |  |  |  |
|  | Falta de aplicabilidad de contenidos de la capacitación en la práctica | 0.651 |  |  |  |  |  |  |  |  |  |
|  | Modalidad presencial de la capacitación en la guía mhGAP | 0.503 |  |  |  |  |  |  |  |  |  |
|  | Expertis de los ponentes de la capacitación en la guía mhGAP | 0.345 |  |  |  |  |  |  |  |  |  |
|  | Modalidad a distancia de la capacitación en la guía mhGAP | 0.302 |  |  |  |  |  |  |  |  |  |
|  | Temas de la capacitación en la guía mhGAP | 0.709 |  |  |  |  |  |  |  |  |  |
| Barreras de la persona atendida | | | | | | | | | | |  |
|  | Dificultad de las personas atendidas para asistir presencialmente al servicio | 0.748 | 29.81 | 0.019 | 16 | 0.978 | 0.962 | 0.083 | 0.041 | 0.904 | |
|  | Dificultad de las personas atendidas para recibir el servicio a distancia | 0.636 |  |  |  |  |  |  |  |  |  |
|  | Nivel educativo de las personas atendidas | 0.689 |  |  |  |  |  |  |  |  |  |
|  | Lengua / idioma de las personas atendidas | 0.544 |  |  |  |  |  |  |  |  |  |
|  | Falta de adherencia a las recomendaciones | 0.850 |  |  |  |  |  |  |  |  |  |
|  | Inasistencias / impuntualidad de las personas atendidas | 0.830 |  |  |  |  |  |  |  |  |  |
|  | Resistencia de las personas atendidas a recibir intervenciones psicosociales | 0.872 |  |  |  |  |  |  |  |  |  |
|  | Resistencia de las personas atendidas a recibir tratamiento farmacológico | 0.722 |  |  |  |  |  |  |  |  |  |
| Barreras del proveedor de tratamiento | | | | | | | | | | |  |
|  | Dificultad para adherirse a los procedimientos de la guía mhGAP | 0.840 | 12.4 | 0.088 | 7 | 0.988 | 0.974 | 0.079 | 0.024 | 0.897 | |
|  | Dificultad para comprender los fundamentos de la guía mhGAP | 0.799 |  |  |  |  |  |  |  |  |  |
|  | Percepción de poca utilidad de la guía mhGAP | 0.736 |  |  |  |  |  |  |  |  |  |
|  | Dificultad para tomar decisiones de derivación | 0.831 |  |  |  |  |  |  |  |  |  |
|  | Escasa supervisión para la implementación de la guía mhGAP | 0.677 |  |  |  |  |  |  |  |  |  |
|  | Dificultad de comunicación con profesionales de la salud de otras disciplinas | 0.709 |  |  |  |  |  |  |  |  |  |
| Frecuencia de implementación general | | | | | | | | | | |  |
|  | Habilidades de comunicación eficaces | 0.536 | 261.08 | <.001 | 9 | 0.92 | 0.90 | 0.105 | 0.058 | 0.940 | |
|  | Promover respeto y dignidad | 0.644 |  |  |  |  |  |  |  |  |  |
|  | Identificar manifestaciones de urgencia (riesgo inminente de autolesión / suicidio, intoxicación, abstinencia o sobredosis, convulsiones) | 0.675 |  |  |  |  |  |  |  |  |  |
|  | Evaluar salud física | 0.535 |  |  |  |  |  |  |  |  |  |
|  | Determinar si existe un trastorno mental, neurológico o por consumo de sustancias | 0.850 |  |  |  |  |  |  |  |  |  |
|  | Elaboración de un plan de tratamiento | 0.757 |  |  |  |  |  |  |  |  |  |
|  | Psicoeducación | 0.925 |  |  |  |  |  |  |  |  |  |
|  | Reducción de estrés | 0.927 |  |  |  |  |  |  |  |  |  |
|  | Fortalecer el apoyo social | 0.874 |  |  |  |  |  |  |  |  |  |
|  | Promover funcionamiento en actividades diarias | 0.875 |  |  |  |  |  |  |  |  |  |
|  | Tratamiento psicológico | 0.700 |  |  |  |  |  |  |  |  |  |
|  | Derivar a la persona a especialista u hospital | 0.716 |  |  |  |  |  |  |  |  |  |
|  | Planear seguimientos | 0.800 |  |  |  |  |  |  |  |  |  |
|  | Trabajar con la familia | 0.728 |  |  |  |  |  |  |  |  |  |
|  | Vincular a la persona con otros servicios (empleo, educación, sociales) | 0.753 |  |  |  |  |  |  |  |  |  |
|  | Atención a grupos especiales (niños y adolescentes, mujeres embarazadas y en lactancia, adultos mayores) | 0.638 |  |  |  |  |  |  |  |  |  |
| Utilidad de implementación general | | | | | | | | | | |  |
|  | Habilidades de comunicación eficaces | 0.797 | 299.46 | <.001 | 90 | 0.93 | 0.91 | 0.117 | 0.039 | 0.960 | |
|  | Promover respeto y dignidad | 0.929 |  |  |  |  |  |  |  |  |  |
|  | Identificar manifestaciones de urgencia (riesgo inminente de autolesión / suicidio, intoxicación, abstinencia o sobredosis, convulsiones) | 0.933 |  |  |  |  |  |  |  |  |  |
|  | Evaluar salud física | 0.726 |  |  |  |  |  |  |  |  |  |
|  | Determinar si existe un trastorno mental, neurológico o por consumo de sustancias | 0.809 |  |  |  |  |  |  |  |  |  |
|  | Elaboración de un plan de tratamiento | 0.913 |  |  |  |  |  |  |  |  |  |
|  | Psicoeducación | 0.892 |  |  |  |  |  |  |  |  |  |
|  | Reducción de estrés | 0.935 |  |  |  |  |  |  |  |  |  |
|  | Fortalecer el apoyo social | 0.917 |  |  |  |  |  |  |  |  |  |
|  | Promover funcionamiento en actividades diarias | 0.904 |  |  |  |  |  |  |  |  |  |
|  | Tratamiento psicológico | 0.773 |  |  |  |  |  |  |  |  |  |
|  | Derivar a la persona a especialista u hospital | 0.828 |  |  |  |  |  |  |  |  |  |
|  | Planear seguimientos | 0.779 |  |  |  |  |  |  |  |  |  |
|  | Trabajar con la familia | 0.828 |  |  |  |  |  |  |  |  |  |
|  | Vincular a la persona con otros servicios (empleo, educación, sociales) | 0.629 |  |  |  |  |  |  |  |  |  |
|  | Atención a grupos especiales (niños y adolescentes, mujeres embarazadas y en lactancia, adultos mayores) | 0.675 |  |  |  |  |  |  |  |  |  |
| Efectividad de la implementación general | | | | | | | | | |  |  |
|  | Habilidades de comunicación eficaces | 0.829 | 291.83 | <.001 | 93 | 0.94 | 0.92 | 0.112 | 0.039 | 0.966 | |
|  | Promover respeto y dignidad | 0.878 |  |  |  |  |  |  |  |  |  |
|  | Identificar manifestaciones de urgencia (riesgo inminente de autolesión / suicidio, intoxicación, abstinencia o sobredosis, convulsiones) | 0.99 |  |  |  |  |  |  |  |  |  |
|  | Evaluar salud física | 0.764 |  |  |  |  |  |  |  |  |  |
|  | Determinar si existe un trastorno mental, neurológico o por consumo de sustancias | 0.780 |  |  |  |  |  |  |  |  |  |
|  | Elaboración de un plan de tratamiento | 0.883 |  |  |  |  |  |  |  |  |  |
|  | Psicoeducación | 0.916 |  |  |  |  |  |  |  |  |  |
|  | Reducción de estrés | 0.926 |  |  |  |  |  |  |  |  |  |
|  | Fortalecer el apoyo social | 0.914 |  |  |  |  |  |  |  |  |  |
|  | Promover funcionamiento en actividades diarias | 0.950 |  |  |  |  |  |  |  |  |  |
|  | Tratamiento psicológico | 0.727 |  |  |  |  |  |  |  |  |  |
|  | Derivar a la persona a especialista u hospital | 0.889 |  |  |  |  |  |  |  |  |  |
|  | Planear seguimientos | 0.794 |  |  |  |  |  |  |  |  |  |
|  | Trabajar con la familia | 0.832 |  |  |  |  |  |  |  |  |  |
|  | Vincular a la persona con otros servicios (empleo, educación, sociales) | 0.852 |  |  |  |  |  |  |  |  |  |
|  | Atención a grupos especiales (niños y adolescentes, mujeres embarazadas y en lactancia, adultos mayores) | 0.664 |  |  |  |  |  |  |  |  |  |
